# Progesterone receptor membrane component 1 promotes the growth of breast cancers by altering the phosphoproteome and augmenting EGFR/PI3K/AKT signalling

**DOI:** 10.1038/s41416-020-0992-6

**Published:** 2020-07-24

**Authors:** Diego A. Pedroza, Venkatesh Rajamanickam, Ramadevi Subramani, Alejandra Bencomo, Adriana Galvez, Rajkumar Lakshmanaswamy

**Affiliations:** 1 Graduate School of Biomedical Sciences, Texas Tech University Health Sciences Center El Paso, El Paso, TX 79905 USA; 2 Earle A. Chiles Research Institute, Providence Cancer Institute, Portland, OR 97213 USA; 3 Department of Molecular and Translational Medicine, Texas Tech University Health Sciences Center El Paso, El Paso, TX 79905 USA

**Keywords:** Breast cancer, Breast cancer

## Abstract

**Background:**

Increased expression of the progesterone receptor membrane component 1 (PGRMC1) has been linked to multiple cancers, including breast cancer. Despite being a regulatory receptor and a potential therapeutic target, the oncogenic potential of PGRMC1 has not been studied.

**Methods:**

The impact of PGRMC1 on breast cancer growth and progression was studied following chemical inhibition and alteration of PGRMC1 expression, and evaluated by using online-based gene expression datasets of human breast cancer tissue. MTS, flow cytometry, qPCR, Western blotting, confocal microscopy and phosphoproteome analysis were performed.

**Results:**

We observed higher PGRMC1 levels in both ER-positive ZR-75-1 and TNBC MDA-MB-468 cells. Both chemical inhibition and silencing decreased cell proliferation, induced cell-cycle arrest, promoted apoptosis and reduced the migratory and invasive capabilities of ZR-75-1 and MDA-MB-468 cells. Further, phosphoproteome analysis demonstrated an overall decrease in activation of proteins involved in PI3K/AKT/mTOR and EGFR signalling pathways. In contrast, overexpression of PGRMC1 in non-malignant MCF10A cells resulted in increased cell proliferation, and enhanced activity of PI3K/AKT/mTOR and EGFR signalling pathways.

**Conclusions:**

Our data demonstrate that PGRMC1 plays a prominent role in regulating the growth of cancer cells by altering the PI3K/AKT/mTOR and EGFR signalling mechanisms in both ER-positive and TNBC cells.

## Background

As the most frequently diagnosed cancer in women worldwide, in 2018, breast cancer accounted for ~2.08 million diagnoses and resulted in over 626,000 deaths.^1^ In the United States, 41,760 women were expected to die from breast cancer in 2019, and diagnosis of 268,600 new cases was projected for the same year.^2^ Treatment options for breast cancer patients depend on the histological grade and expression status of the oestrogen receptor (ER), progesterone receptor (PR) and human epidermal growth factor receptor 2 (HER2/neu). ER-positive breast cancers are mainly treated via targeted endocrine therapy.^3,4^ HER2-/neu-overexpressing breast cancer patients are treated with the monoclonal antibody trastuzumab.^5^ Generally, chemotherapy and/or radiation is recommended for triple-negative breast cancers (TNBCs).^6^ However, it is well-known that many patients demonstrate intrinsic or de novo resistance to these therapies.^7–9^ Consequently, it is necessary to identify novel therapeutic targets.

Progesterone receptor membrane component 1 (PGRMC1)—a membrane-bound protein containing an N-terminal transmembrane segment and a C-terminal cytochrome b5-like/steroid- binding domain—is encoded by the PGRMC1 gene. It is a member of the membrane-associated progesterone receptor (MAPR) family of proteins.^10,11^ PGRMC1 has been linked to multiple cancers, including ovarian,^12^ lung^13^ and breast cancer.^14^ PGRMC1 expression is observed in primary breast tumour tissues, and its expression was found to correlate with larger tumour size and lymph node metastasis.^15^ Moreover, in extant studies, PGRMC1 was demonstrated to enhance the chemotherapeutic resistance of several cancers, including breast cancer.^16^ These observations indicate that patients in whom PGRMC1 is overexpressed would have poor disease-free and overall low survival rates. In colon cancer cells, the interaction between PGRMC1 and drug-metabolising cytochrome P450 enzymes facilitates doxorubicin degradation.^10^ Further, PGRMC1 has been linked to epithelial-to-mesenchymal transition (EMT), motility, anchorage-independent growth, angiogenesis and overall metastasis in multiple cancer cell lines.^17^ Interestingly, PGRMC1 has been demonstrated to specifically interact and modulate the epidermal growth factor receptor (EGFR) levels in multiple tumour cells.^18^ Anti-EGFR treatments, such as those involving monoclonal antibodies and small-molecule tyrosine kinase inhibitors, are currently offered to patients diagnosed with specific types of cancers.^19^ However, no EGFR-targeted breast cancer treatment is presently available.^19^ While EGFR-mediated signalling has been extensively studied, PGRMC1 signalling and interactions with EGFR control mechanisms remain insufficiently understood.

In the current study, we found that PGRMC1 is overexpressed in both ER-positive and TNBC cells. As part of our investigations, we observed that both chemical inhibition and silencing of PGRMC1 reduce cell proliferation, induce apoptosis, cause cell-cycle arrest and inhibit invasion and migration of breast cancer cells. Mechanistically, we demonstrate that PGRMC1 influences breast tumorigenesis through the PI3K/AKT/mTOR and EGFR signalling pathway. We, therefore, posit that PGRMC1 plays a crucial role in breast cancers, and could potentially serve as a target for both ER-positive and TNBC cells.

## Methods

### Cell lines and cell culture

MCF12A, MCF10A, MCF7, T47D, ZR-75-1, MDA-MB-231 and MDA-MB-468 were acquired from the American Type Culture Collection (ATCC, Manassas, VA, USA). MCF7, T47D and ZR-75-1 were cultured in phenol red-free RPMI-1640 media supplemented with 10% foetal bovine serum (FBS), 100 units/mL penicillin and 100 μg/mL streptomycin. MDA-MB-231 and MDA-MB-468 were cultured in RPMI-1640 supplemented with 10% foetal bovine serum (FBS), 100 units/mL penicillin and 100 μg/mL streptomycin. MCF12A cells were cultured in DMEM/F12 with 5% horse serum, and supplemented with SingleQuots^TM^ (Lonza, Portsmouth, NH, USA, Cat No: CC-4133). MCF10A cells were cultured in mammary epithelial basal media (MEBM) supplemented with SingleQuots^TM^ (Lonza, Portsmouth, NH, USA, Cat No: CC-4133). All cells were maintained in 5% CO_2_ at 37 °C.

### RNA extraction and real-time quantitative RT-PCR

Total RNA was isolated from the cells using TRIzol reagent (Life Technologies, Cat. No. 15596-018). cDNA for gene expression analysis was prepared using the RT2 first-strand kit (Qiagen, Cat. No. 330401). qRT-PCR was performed in triplicates using the Quantitech SYBR green kit (Qiagen, Cat. No. 204141) in a StepOnePlus real-time PCR system (Applied Biosystems, Foster City, CA, USA). Analyses were performed based on the comparative Ct method (2−ΔΔCt) with GAPDH as the housekeeping reference gene. The primers utilised were as follows:

PGRMC1 Forward 5′-CGACGGCGTCCAGGACCC-3′

reverse 5′-TCTTCCTCATCTGAGTACACAG-3′

GAPDH Forward 5′-CAGCCTCAAGATCATCAGCAATGC-3′

reverse 5′-AGACCACCTGGTGCTCAGTGTAG-3′.

### Silencing PGRMC1

siRNAs targeting PGRMC1 and control scrambled-sequence siRNA were purchased from Origene (Rockville, MD, USA). Using MIrus bio TransIT siQUEST transfection reagent (Mirus Bio, Madison, WI, USA), 5 × 10^5^ cells were transfected with different siRNA concentrations and sequences (A, B and C) ranging from 20 to 60 nM for 48 h. In accordance with the manufacturer’s protocol, the ratio of transfection reagent to siRNA was maintained at 1:1 to ensure efficient silencing while minimising toxicity. Western blot analysis was performed to confirm efficient silencing of PGRMC1. PGRMC1 siRNA sequences utilised were as follows:

SR323253A-rGrArUrCrArArCrUrUrUrUrArGrUrCrArUrGrArUrGrUrUCT

SR323253B-rCrArArUrUrGrArCrUrUrArArCrUrGrCrArUrGrArUrUrUCT

SR323253C-rUrCrArArCrUrUrUrUrArGrUrCrArUrGrArUrGrUrUrCrUGT.

### PGRMC1 overexpression

Cells were seeded in a six-well plate at a density of 5 × 10^5^ cells/well and were transfected after 24 h with PGRMC1 control pCMV-XL6 and pCMV6-AC-GFP plasmids (Origene). Cells were transfected using Mirus bio TransIT 2020 transfection reagent (Mirus Bio) as per the manufacturer’s protocol. PGRMC1 overexpression was confirmed by Western blot.

### Cell-proliferation assay

Cells were seeded in 96-well plates in six experimental replicates at a density of 0.6 × 10^4^ cells/well and in 24-well plates in triplicates at a density of 1 × 10^5^ cells/well and incubated for 24 h under 5% CO_2_ at 37 °C before being transfected with PGRMC1 siRNA or treated with PGRMC1 antagonist AG-205 at varying doses (10, 20, 30, 40, 50, 75 and 100 μM). Using MTS (3-(4,5-dimethylthiazol-2-yl)-5-(3-carboxymethoxyphenyl)-2-(4-sulfophenyl)-2H-tetrazolium) assay, cell proliferation (96-well plates) was measured λ = 490 nm using a microplate reader (CLARIOstar, BMG LABTECH). Using a haemocytometer (24-well plates), cell counting was assessed following trypan blue staining. Both proliferation assays were performed following 24 h of AG-205 treatment and time-dependent PGRMC1 siRNA transfection.

### Immunofluorescence

Cells were fixed in 4% paraformaldehyde, permeabilised with 0.2% Triton X-100 and blocked with 5% BSA for 1 h. They were subsequently incubated with the corresponding primary antibody for 1 h, followed by incubation with Alexa Fluor 488-conjugated goat anti-rabbit secondary antibody (Life Technologies, Grand Island, NY) for 1 h. The Nikon Eclipse Ti confocal laser-scanning microscope (Nikon Instruments, Inc., Melville, NY, USA) was used to capture the images. Expression of cleaved-caspase 3, PGRMC1 and EGFR in ZR-75-1 and MDA-MB-468 was measured 24 h after AG-205 treatment (50 μM dose) and 48 h post PGRMC1 siRNA transfection.

### Western blot analysis

Following PGRMC1 siRNA transfection and AG-205 treatment, cells were harvested, and protein was extracted using the mammalian Protein Extraction Reagent (mPER) according to the manufacturer’s protocol (Thermo Scientific, US). Protein concentration was quantitated and determined using the Pierce^TM^ BCA Protein Assay Kit (Thermo Scientific, US). Equal amounts of protein were loaded and separated by SDS-PAGE using Mini-Protean TGX polyacrylamide gels (Bio-Rad). Proteins were then transferred onto PVDF membranes, blocked with 5% BSA in 1 × TBST and probed with the respective primary antibodies followed by the appropriate secondary antibody, and were subsequently visualised using an enhanced chemiluminescence kit (Thermo Scientific, US). Primary antibodies used for the experiments were as follows: AKT (14702S), p-AKT (4060S), p-EGFR (3777S), mTOR (2972S), p-mTOR (2971S), PGRMC1 (13856S), Cl. Caspase 3 (9661S), Cl. PARP (5625S), PARP (9532S), BAX (2772S), CDK4 (12790P) and Cyclin D1 (2978P), all of which were obtained from Cell Signaling Technology; PTEN (sc-7974) and p-EGFR (sc-101665), purchased from Santa Cruz Biotechnology; EGFR (ab-2430-1) from Abcam; β-actin (A1978) from Sigma-Aldrich. ImageJ software was used to quantify protein levels detected.

### Apoptosis analysis and flow cytometry

Apoptosis was analysed using Annexin V-FITC Apoptosis Detection Kit I according to the manufacturer’s instructions bioLegened^TM^. ZR-75-1 and MDA-MB-468 cells were seeded in six-well plates at a density of 5 × 10^5^ cells/well and were treated with 50 μM AG-205 for 24 h or transfected with PGRMC1 siRNA for 48 h. Apoptosis was studied using FACS Accuri C6 flow cytometer (San Jose, CA, USA).

### Invasion/migration assay

Invasive and migratory capabilities of ZR-75-1 and MDA-MB-468 cells following 50 μM AG-205 treatment or PGRMC1 siRNA transfection were assessed using the invasion and migration assay. For invasion, Matrigel (Collaborative biomedical products, Bedford, MA, USA) was placed onto 8.0- μm pore Transwell chambers (Corning Incorporated, Corning, NY, USA) and was allowed to polymerise for 4 h at 37 °C. Medium containing 10% FBS was added to the bottom of the 24-well plate. Next, Transwell chambers were placed in 24-well plates, and 5 × 10^5^ cells were seeded on top of the Matrigel and were incubated for 24 h at 37 °C. For migration, serum-free medium was placed onto 8.0-μm pore Transwell chambers (Corning Incorporated, Corning, NY, USA), and medium containing 10% FBS was added to the bottom of the wells. Transwell chambers were then placed in 24-well plates, and 5 × 10^5^ cells were seeded on top with serum-free media and were incubated for 24 h at 37 °C. Cells that invaded/migrated to the lower chamber were fixed with 5% formalin and stained with 0.2% crystal violet. The stained cells were counted in three distinct random fields; the counts were averaged and plotted.

### Cell-cycle analysis

Cell-cycle analysis was conducted by treating ZR-75-1 and MDA-MB-468 cells with 50 μM AG-205 for 24 h or PGRMC1 silencing for 48 h followed by fixation in 70% ethanol for 1 h. Cells were subsequently treated with RNAse and incubated for 30 min at 4 °C, after which DNA content was measured by propidium iodine (PI) staining. The analysis was performed using FACS Accuri C6 flow cytometer (San Jose, CA, USA).

### In silico analysis and bioinformatics

Online RNAseq-based gene expression analysis findings pertaining to PGRMC1 in breast cell lines and breast tumour datasets were downloaded from the UCSC Xena browser (https://xenabrowser.net) obtained from Heiser RNAseq data (18,632 genes in 54 breast cancer cell lines) and TCGA Breast Cancer data (PAM50 subtype RNAseq TCGA AWG data from 1,247 tumour tissues). PGRMC1 protein expression levels in non-malignant and malignant breast tissue samples from the Human Protein Atlas database (http://www.proteinatlas.org/) were also examined. For the present study, bespoke proprietary software was developed using Python 3.0 platform to compute and process the raw fluorescent intensities for proteins in total and phosphorylated form. Differential expression analyses were conducted, and heat maps were generated using open-source R libraries. Moreover, gene set enrichment analysis was conducted using gprofiler, whereby the genes were ranked by effect size. Gprofiler maps ranked genes with respect to known functional information databases and pathways. The findings revealed statistically significantly enriched terms and pathways, as inferred by gprofiler. Finally, the top 20 differentially expressed genes were subjected to network analysis using genemania 3.

### Phospho-explorer antibody array

The glass-slide-based phospho-explorer antibody array was utilised according to the manufacturer’s instructions (Full Moon BioSystems Inc.). ZR-75-1 and MDA-MB-468 cells treated with 50 μM AG-205 and PGRMC1 siRNA transfected were harvested using protein extraction buffer (Full Moon BioSystems Inc.) diluted in labelling buffer and mixed with Biotin/DMF labelling solution. Biotinylated proteins were incubated on individual antibody-coated slides, and were labelled with Cy3-streptavidin (GE Healthcare). To detect fluorescent intensity, the slides were scanned using an Axon GenePix Array Scanner (Molecular Devices). The intensity values obtained were normalised relative to internal controls and analysed.

### Statistical analyses

Samples used for data collection were obtained via three independent experiments, and the findings are presented as mean ± SD. Statistical analysis was conducted using GraphPad Prism 7 software, version 7.0 (GraphPad Prism Software, San Diego, CA, USA) whereby *P < 0.05 was considered statistically significant.

## Results

### PGRMC1 is overexpressed in breast cancers

**Fig. 1:**
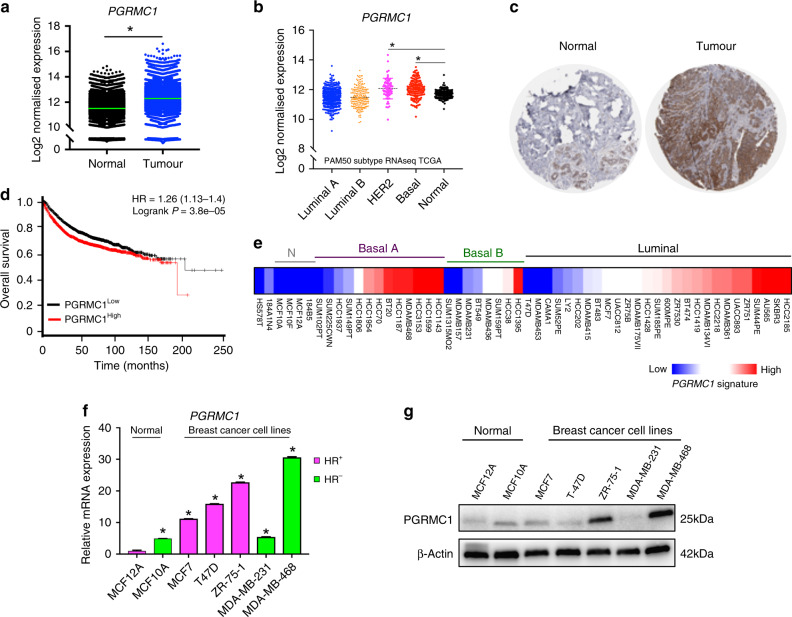
PGRMC1 is highly expressed in ER-positive and TNBCs. **a** Analysis of PGRMC1 expression in GTEX normal (*n* = 7,824) breast tissue vs TCGA tumour (*n* = 10,534) breast tissue. **b** Subgroup analysis classified by PAM50 subtypes Luminal A (*n* = 434), Luminal B (*n* = 194), HER2 (*n* = 67), Basal (*n* = 142) and Normal (*n* = 119) from RNAseq TCGA-AW datasets of PGRMC1 in human breast tissues (*n* = number of tissues). **c** PGRMC1 expression in normal and breast tumour tissue by IHC obtained from the HUMAN PROTEIN ATLAS. **d** Overall survival of breast cancer patients with low and high PGRMC1 levels is demonstrated using the online tool. **e** Heatmap depicting PGRMC1 relative expression in 54 breast cell lines using Heiser RNAseq data. **f** PGRMC1 relative mRNA expression generated by qPCR in a panel of normal and breast cancer cell lines. **g** PGRMC1 protein expression in a panel of normal and breast cancer cell lines by western blot analysis. Analysis of (**a**, **b**) is calculated using *t* test comparing normal vs tumour tissue, *P < 0.05. Analysis of (**f**) is the mean ± SD of three independent experimental replicates (*n* = 3*;* *P < 0.05 calculated using one-way ANOVA, multiple comparisons).

We studied PGRMC1 expression in human tumour tissue by comparing the genotype-tissue expression (GTEx) portal data pertaining to normal breast tissue and The Cancer Genome Atlas (TCGA). The downloaded data showed a significant increase in PGRMC1 expression in breast tumour tissues compared with normal tissues (Fig. 1a). By further examining the TCGA datasets, we observed increased PGRMC1 expression in HER2 and basal-like subtypes compared with normal breast tissue (*n* = 1,247), as shown in Fig. 1b. Using the information sourced from the Human Protein Atlas database (http://www.proteinatlas.org/), we observed strong PGRMC1 staining by immunohistochemistry (IHC) in breast ductal carcinoma compared with normal breast tissue (Fig. 1c). Clinical relevance of PGRMC1 expression and survival obtained through Kaplan–Meier plotter (KM plotter) tool (http://kmplot.com/analysis) demonstrated positive association between high PGRMC1 mRNA expression in breast cancer patients and significantly poorer survival probability (*P* = 3.8e−05) compared with patients in whom PGRMC1 mRNA expression is low (Fig. 1d). Once high PGRMC1 expression in breast cancers was established, the investigation shifted towards elucidating the mechanisms behind this phenomenon. For this purpose, multiple breast cancer cells were screened for PGRMC1 expression, which was analysed using online RNAseq-based gene expression from non-malignant and malignant breast cell lines (Breast Cancer Cell Lines-Heiser 2012 dataset). Our analysis revealed PGRMC1 to be widely expressed throughout multiple subtypes of breast cancer cells and tissues (Fig. 1e and Supplementary Fig. 1a–c). PGRMC1 overexpression was further validated by analysing a panel of proprietary normal breast and breast cancer cell lines, which revealed high mRNA and protein levels of PGRMC1 in both ER-positive ZR-75-1 and TNBC MDA-MB-468 cell lines (Fig. 1f, g). These observations point to an association between PGRMC1 and both ER-positive and TNBC cells, as well as with poor survival prognosis.

### PGRMC1 signal disruption inhibits ER-positive and TNBC cell growth and survival

**Fig. 2:**
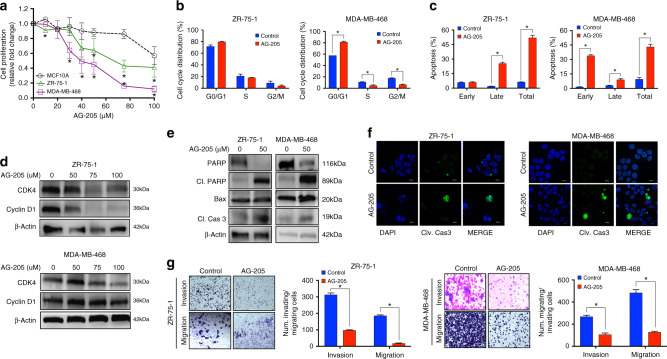
AG-205 selectively inhibits ER-positive and TNBC cell growth and survival. **a** Dose-dependent cell proliferation by MTS assay of normal MCF10A breast cells, ZR-75-1 and MDA-MB-468 breast cancer cells following 10, 20, 30, 40, 50, 75 and 100 μM AG-205 treatment for 24 h. **b** Cell-cycle analysis by flow cytometry following 50 μM AG-205 treatment in ZR-75-1 and MDA-MB-468 cells. **c** Apoptosis analysis by flow cytometry following 50 μM AG-205 treatment in ZR-75-1 and MDA-MB-468 cells. **d** Western blot analysis of important cell-cycle regulators, CDK4 and Cyclin D1 following different concentrations 50, 75 and 100 μM AG-205 treatment in ZR-75-1 and MDA-MB-468 cells. **e** Protein levels of apoptotic-associated markers, PARP, Cleaved PARP, Bax and Cleaved-caspase 3 following 50 μM AG-205 treatment in ZR-75-1 and MDA-MB-468 cells by western blot analysis. **f** Immunofluorescence depicting activated cleaved-caspase 3 following 50 μM AG-205 treatment in ZR-75-1 and MDA-MB-468 cells (×100 magnification). **g** Transwell invasion and migration assays of ZR-75-1 and MDA-MB-468 cells following 50 μM AG-205 treatment. Analysis of **a** is the mean ± SD of three independent experimental replicates (*n* = 3) *P < 0.05 calculated using *t* test comparing normal MCF10A breast cells with ZR-75-1 and MDA-MB-468 breast cancer cells. Analysis of (**b**, **c**, **g**) is the mean ± SD of three independent experimental replicates (*n* = 3). *P < 0.05 as compared with control.

Normal MCF10A breast cells, as well as ZR-75-1 and MDA-MB-468 breast cancer cells, were treated with a selective PGRMC1 inhibitor (AG-205) at different concentrations (10–100 μM) for 24 h to determine the optimal dose for studying the PGRMC1 mechanism of action. Our observations revealed that AG-205 decreased proliferation of both breast cancer cell types in a dose-dependent manner, while exhibiting minimal effects on normal breast cells (Fig. 2a and Supplementary Fig. 2a–c). Interestingly, increased AG-205 concentration yielded minimal alterations in PGRMC1 protein levels in both breast cancer cell lines (Supplementary Fig. 3a, b). These findings suggested that AG-205 acts as a PGRMC1 signalling inhibitor, rather than a protein downregulator. Based on our results, 50 μM was adopted for further experiments, due to its ability to decrease proliferation of both breast cancer cell lines by ~40–50%. Next, we investigated cell- cycle progression and apoptosis following AG-205 treatment. In both breast cancer cell lines, PGRMC1 signal inhibition by AG-205 induced G1-phase arrest and decreased both S and G2/M phases, although differences were statistically significant in MDA-MB-468 cells only (Fig. 2b and Supplementary Fig. 4a). AG-205 also significantly induced apoptosis in ZR-75-1 (by 52.3%) and MDA-MB-468 (by 44.3%) breast cancer cell lines (Fig. 2c and Supplementary Fig. 4c). Levels of CDK4 and Cyclin D1, both of which are important molecular markers associated with cell-cycle progression, decreased following AG-205 treatment in both breast cancer cell lines (Fig. 2d and Supplementary Fig. 5a, b). Moreover, pro-apoptotic proteins cleaved PARP, Bax and cleaved-caspase 3 were increased in both cell lines following AG-205 treatment (Fig. 2e and Supplementary Fig. 6a, b). Immunofluorescence (IF) analysis of cleaved-caspase 3 in both breast cancer cell lines further confirmed cell death by apoptosis (Fig. 2f). Furthermore, AG-205 significantly inhibited the migratory and invasive capabilities of both ZR-75-1 and MDA-MB-468 cells (Fig. 2g).

### Silencing PGRMC1 disrupts breast cancer cell growth and survival

**Fig. 3:**
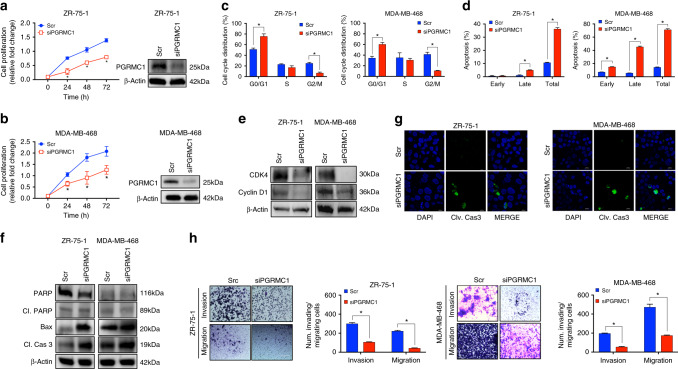
Silencing PGRMC1 inhibits growth and survival of breast cancer cells. **a**, **b** Time-dependent cell proliferation by MTS following silencing of PGRMC1 by siRNA and protein levels by western blot analysis of PGRMC1 to confirm effective PGRMC1 silencing in ZR-75-1 and MDA-MB-468 cells. **c** Cell-cycle analysis by flow cytometry following PGRMC1 silencing in ZR-75-1 and MDA-MB-468 cells. **d** Apoptosis analysis by flow cytometry following PGRMC1 silencing in ZR-75-1 and MDA-MB-468 cells. **e** Protein levels of cell-cycle regulators, CKD4 and Cyclin D1, following PGRMC1 silencing in ZR-75-1 and MDA-MB-468 cells by western blot analysis. **f** Protein levels of apoptotic-associated markers, PARP, Cleaved PARP, Bax and Cleaved-caspase 3, following PGRMC1 silencing in ZR-75-1 and MDA-MB-468 cells by western blot analysis. **g**. Immunofluorescence depicting activated cleaved-caspase 3 following PGRMC1 silencing in ZR-75-1 and MDA-MB-468 cells (×100 magnification). **h** Transwell invasion and migration assays of ZR-75-1 and MDA-MB-468 following silencing of PGRMC1. Analysis of **a**, **b** is the mean ± SD of three independent experimental replicates. (*n* = 3) *P < 0.05 calculated using *t* test comparing Scramble (Scr) to siPGRMC1. Analysis of **c**, **d**, **h** is the mean ± SD of three independent experimental replicates (*n* = 3). *P < 0.05 as compared with control.

Utilising siRNA gene silencing technology, effective silencing of PGRMC1 in ZR-75-1 and MDA-MB-468 was achieved, which in turn decreased cell proliferation of both ER-positive and TNBC cells (Fig. 3a, b and Supplementary Fig. 7a, b). Further, significant alterations in cell-cycle progression were observed after PGRMC1 silencing, as cell cycle in both breast cancer cell lines was arrested in the G1 phase (Fig. 3c and Supplementary Fig. 4b). PGRMC1 silencing also induced apoptosis in ZR-75-1 (by 36%) and MDA-MB-468 (by 71%) cell lines (Fig. 3d and Supplementary Fig. 4d). Levels of molecular markers associated with cell-cycle progression, CDK4 and Cyclin D1, also decreased following PGRMC1 silencing (Fig. 3e and Supplementary Fig. 8a, b), whereas the expression of pro-apoptotic markers, Bax and cleaved-caspase 3, increased in both breast cancer cell lines (Fig. 3f and Supplementary Fig. 9a, b). The activation of cleaved-caspase 3 was also confirmed by IF (Fig. 3g). Silencing PGRMC1 also impaired the migratory and invasive capabilities of both breast cancer cell lines (Fig. 3h, i). These findings signify that PGRMC1 plays a major role in the growth and progression of breast cancers.

### PGRMC1 disruption influences the phosphoproteome of breast cancers

**Fig. 4:**
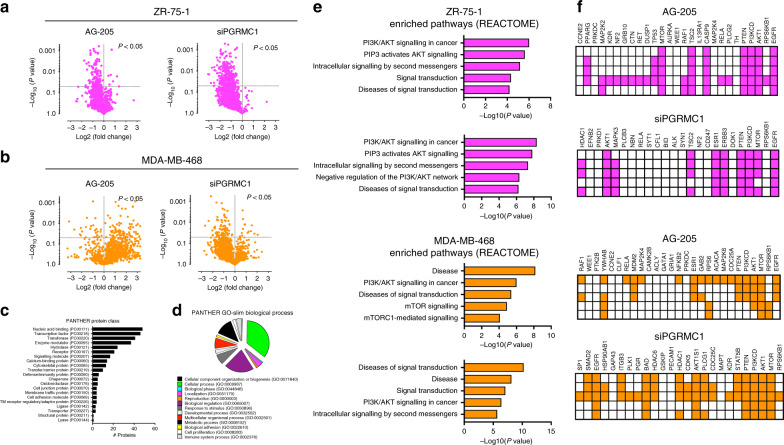
Phosphoproteome analysis connects PGRMC1 signalling to breast cancer survival pathways. **a**, **b** Volcano plots obtained from phosphoproteome analysis representing both total and phosphorylated protein forms following 50 μM AG-205 treatment or PGRMC1 silencing in ZR-75-1 and MDA-MB-468 cells. **c** Protein classes involved in the phosphoproteome analysis generated by PANTHER Protein Class subsets. **d** Gene ontology (GO) classification of biological processes involved in the phosphoproteome analysis generated by PANTHER GO-Slim Biological Process subsets. **e**, **f** Rows represent the top five GO term- enriched altered signalling pathways analysed by REACTOME and heatmap clusters of differentially expressed  genes identified by phosphoproteome analysis following either 50 μM AG-205 treatment or PGRMC1 silencing. Pink represents pathways and genes associated with ZR-75-1, and orange represents pathways and genes associated with MDA-MB-468 cells.  Volcano plots: significant down- and upregulated proteins are represented, *P* < 0.05.

Changes in the PGRMC1 signalling mechanism were next investigated utilising a chip-based phosphoprotein array, which is designed to qualitatively profile 1,318 total and site-specific phosphorylated proteins. As a part of this work, we assessed changes in protein modifications following PGRMC1 signal inhibition by AG-205 or PGRMC1 silencing in both ZR-75-1 and MDA-MB-468 breast cancer cell lines (Fig. 4a, b). Examination of PANTHER protein-class datasets revealed changes in multifunctional proteins, including transcription factors, signalling molecules and membrane traffic proteins (Fig. 4c). Moreover, PANTHER GO-Slim Biological datasets demonstrated changes in involvement of cellular component organisation, biogenesis and cell-proliferation processes^20^ (Fig. 4d). Gene ontology (GO) term enrichment pathway analysis by REACTOME revealed alterations to important signalling mechanisms following AG-205 treatment or PGRMC1 silencing of both breast cancer cell lines (Fig. 4e). Overlapping significant changes to GO terms for both cell lines included PI3K/AKT signalling in cancer, signal transduction, diseases of signal transduction and signalling by receptor tyrosine kinases (Fig. 4e). Pink is associated with ZR-75-1, and orange represents pathways and genes associated with MDA-MB-468 cells. The enriched pathways correlated with commonly differentially expressed genes from the array, including *PTEN, PIK3CD, AKT1, mTOR, RPS6KB1* and *EGFR*, throughout the entire groups (Fig. 4f and dataset 1 and dataset 2). Network analysis demonstrated the top 20 altered genes following AG-205 treatment and PGRMC1 silencing (Supplementary Fig. 10a and Supplementary Fig. 11a). Following PGRMC1 signal inhibition or silencing, the data generated associate cell survival mechanisms with PI3K/AKT/mTOR and EGRF signalling.

### PGRMC1 disruption alters phosphorylation sites of cell survival mechanisms

**Fig. 5:**
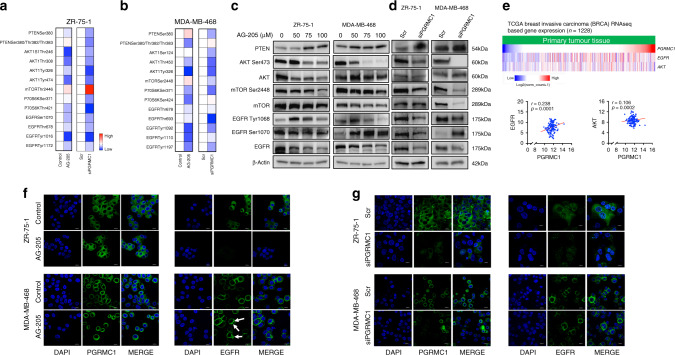
Inhibition of PGRMC1 disrupts AKT/mTOR and EGFR phosphorylation sites. **a**, **b** Heat maps depicting increased or decreased phosphorylation at multiple sites of PTEN, AKT, mTOR, P70S6K and EGFR in ZR-75-1 following 50 μM AG-205 treatment and PGRMC1 silencing in ZR-75-1 and MDA-MB-468 cells. **c**, **d** Protein levels of PTEN, AKT, mTOR, EGFR and their phosphorylation sites following 0, 50, 75 and 100 μM AG-205 treatment or PGRMC1 silencing by western blot analysis. e. Positive correlation between *PGRMC1*, *EGFR* and *AKT* in 1 228 primary tumour tissues from BRCA TCGA database. **f**, **g** Immunofluorescence depicting PGRMC1 and EGFR expression in ZR-75-1 and MDA-MB-468 cells following 50 μM AG-205 treatment or PGRMC1 silencing (×100 magnification). Spearman’s test was used to assess correlations.

Given that both PGRMC1 signal disruption and silencing downregulate proteins associated with cell proliferation and cell survival, we further studied the phosphorylation sites of AKT, mTOR, P70S6K and EGFR. The heat maps generated for this purpose demonstrate decreased phosphorylation levels of AKT at Ser124, Tyr450, Tyr326, Thr246 and Tyr474, phosphorylation of mTOR at Thr2446 and Ser2448, phosphorylation of P70S6K at Ser371, Thr421 and Ser424 and phosphorylation of EGFR at Thr678, Tyr1016, Thr1172, Thr693, Tyr1092, Tyr1110 and Tyr1197, in both cell lines after treatment of AG-205 or PGRMC1 silencing (Fig. 5a, b). Further, western blot revealed that AG-205 and PGRMC1 silencing resulted in increased PTEN expression and decreased phosphorylation of AKT (Ser473) and mTOR (Ser2448) (Fig. 5c, d). Interestingly, both total and phosphorylation of EGFR at Tyr1068 decreased in both cell lines, while phosphorylation of EGFR at Ser1070 increased following both AG-205 treatment and PGRMC1 silencing in MDA-MB-468 cells (Fig. 5c, d). This finding is of particular interest because EGFR phosphorylation at Ser1070 plays a critical role in EGFR desensitisation, internalisation and degradation.^21,22^ To further study the role of PGRMC1 in breast cancers, we analysed clinical data from TCGA–BRCA datasets (https://xenabrowser.net). In particular, we identified expression of *EGFR* and *AKT* to be positively correlated with *PGRMC1* gene expression from human breast tissue (Fig. 5e). We next utilised IF to investigate the expression and localisation of PGRMC1 and EGFR following AG-205 treatment and PGRMC1 silencing in both cell lines. IF data demonstrated that AG-205 treatment did not produce any significant change to PGRMC1 expression in either cell line (Fig. 5f). However, EGFR expression was drastically decreased in ZR-75-1 following both AG-205 and PGRMC1 silencing (Fig. 5f, g). Interestingly, in MDA-MB-468 cells, EGFR expression was detected in the plasma membrane and cytoplasm following AG-205 and PGRMC1 silencing (Fig. 5f, g). These findings indicate that AG-205 treatment as well as PGRMC1 silencing impairs important cell-proliferative signalling mechanisms by disrupting the PGRMC1/EGFR axis as phosphorylation of EGFR Ser1070 destabilises, internalises and downregulates EGFR expression.^18,21,22^

### PGRMC1 overexpression causes changes to the phosphoproteome and promotes cell survival

**Fig. 6:**
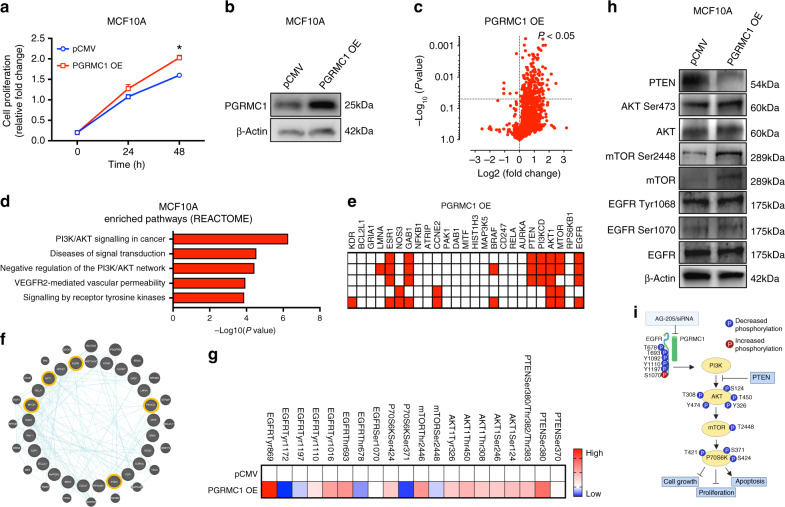
PGRMC1 overexpression alters the phosphoproteome and promotes cell survival of normal breast cells. **a** Time-dependent cell proliferation by MTS assay of PGRMC1-overexpressing MCF10A cells compared with cells transfected with a pCMV empty vector. **b** Protein levels of normal MCF10A breast epithelial cells transfected with pCMV or PGRMC1-overexpressing plasmid by western blot analysis. **c** Volcano plots generated from phosphoproteome analysis representing both total and phosphorylated protein forms of MCF10A-overexpressing cells compared with pCMV-transfected MCF10A cells. **d**, **e** GO term-enriched pathway analysis by REACTOME and heatmap clusters of differentially expressed  genes identified by phosphoproteome analysis following PGRMC1 overexpression in MFC10A cells. **f** Cluster network analysis of the top 20 differentially expressed genes. **g** Heat maps depicting increased or decreased phosphorylation at multiple sites of PTEN, AKT, mTOR, P70S6K and EGFR of MCF10A PGRMC1-overexpressing cells. **h** Protein levels of proliferative markers PTEN, AKT, mTOR, EGFR and their phosphorylation sites in MCF10A pCMV and MCF10A PGRMC1-overexpressing cells by western blot analysis. **i** Schematic of downstream phosphorylation activity following AG-205 treatment or silencing of PGRMC1 of breast cancer cells. Analysis of **a** is the mean ± SD of three independent experimental replicates (*n* = 3). *P < 0.05 calculated using *t* test comparing pCMV with PGRMC1 OE. Volcano plot: significant down- and upregulated proteins are represented, *P* < 0.05.

To further elucidate the PGRMC1 signalling mechanism, PGRMC1 was overexpressed in non-malignant MCF10A breast cells, which resulted in increased cell proliferation (Fig. 6a, b). Following successful overexpression (OE), we performed phosphoproteome analysis, which revealed significant upregulation of phosphorylated proteins (Fig. 6c). Furthermore, REACTOME GO enrichment pathway analysis showed alterations to multiple signalling pathways (Fig. 6d). These pathways correlate with differentially expressed genes, including *GAB1, PTEN, PI3K3CD, AKT1, mTOR* and *EGFR* (Fig. 6e). Network analysis was subsequently performed; the 20 genes that were enriched following PGRMC1 OE were identified (Fig. 6f). These genes exhibited strong interactions with important cell-proliferative markers, including *EGFR, PIK3CD, PTEN, mTOR* and *AKT1* (Fig. 6f). Next, we performed phosphoproteome analysis to further investigate the molecular signalling pathways associated with PGRMC1 OE (Fig. 6g and dataset 3). The resulting heat maps depict differential phosphorylation expression, whereby increased phosphorylation of AKT at Ser124, Ser246, Thr308, Thr450 and Tyr326, phosphorylation of mTOR at Ser2448 and Thr2446, phosphorylation of P70S6K at Ser424 and phosphorylation of EGFR at Thr693, Tyr1016, Tyr1110 and Tyr869 was observed (Fig. 6g). Finally, using Western blot, we confirmed that PGRMC1 OE increases AKT phosphorylation at Ser473, phosphorylation of mTOR at Ser2448 and phosphorylation of EGFR at Tyr1068, accompanied by a decrease in PTEN (Fig. 6h). These results support our assertion that PGRMC1 regulates EGFR, and that AKT and mTOR are downstream targets, whereas PTEN inhibits PGRMC1 downstream signalling in breast cancers (Fig. 6i).

## Discussion

As a part of the present study, we determined that PGRMC1 specifically promotes breast cancer growth by activating the PI3K/AKT/mTOR and EGFR signalling pathway. Utilising a chemical inhibitor and RNA interference of PGRMC1, we studied the phosphoproteome and identified multiple alterations to phosphorylation of key proteins involved in an array of signalling pathways. Our results thus enhance the current understanding of PGRMC1 downstream signalling and broaden the current knowledge of the role of PGRMC1 signalling network in inducing breast cancer cell growth. Moreover, patients in whom high PGRMC1 expression is detected have been found to have lower overall survival rates compared with those in whom PGRMC1 expression is low, suggesting that PGRMC1 contributes to breast cancer aggressiveness, leading to increased mortality rates.^15,23^ However, it has been reported that PGRMC1 mRNA levels decrease as breast cancer patients get older.^24^ Increased levels of PGRMC1 in multiple breast cancer cell lines, including MDA-MB-231, have been previously demonstrated.^16,25^ We noted decreased PGRMC1 expression in MDA-MB-231 breast cancer cells that had previously been utilised to study PGRMC1.^16,26^ Further, Ahmed et al. demonstrated the importance of PGRMC1 in A549 non-small-cell lung cancer cells, and gave a glimpse into a possible role for PGRMC1 in MDA-MB-468 breast cancer cells.^27^ However, because the levels of PGRMC1 have not been demonstrated in MDA-MB-468 or in non-malignant breast cells, we demonstrate high expression of PGRMC1 mRNA and protein in ZR-75-1 and MDA-MB-468, and low expression in MCF10A cells to appropriately study PGRMC1 that correlates with online RNAseq and microarray datasets.

To investigate the PGRMC1 signalling mechanism implicated in breast cancer growth and progression, we used the chemical inhibitor AG-205; this aromatic compound has high-affinity binding to PGRMC1 and is able to disrupt the PGRMC1–heme complex axis as shown by spectroscopic properties.^28^ AG-205 has also been shown to inhibit PGRMC1’s downstream signalling.^27,29^ Findings yielded by our studies demonstrate that disrupting PGRMC1 signalling by AG-205 promotes apoptosis, whereas it inhibits the cell cycle, migratory and invasive capabilities of both ER-positive and TNBC cell lines. Interestingly, AG-205 has been shown to disrupt the interaction between PGRMC1 and actin cytoskeleton-associated proteins; this could have a profound impact on cancer cell metastasis, and why it impaired the migratory capabilities in our study.^30^ Since AG-205 did not decrease PGRMC1 expression, while inducing potent anticancer effects, further analyses were conducted to ascertain that all these effects were due to PGRMC1. For this purpose, PGRMC1 was silenced using RNA interference, yielding similar results to those produced by AG-205, whereby decreased proliferation, increased apoptosis, cell-cycle arrest and inhibition of migration/invasion of breast cancer cells was observed.

PGRMC1 has been shown to be involved in the activation of multiple metabolic pathways that are essential for the growth and survival of malignant cells.^31^ In our study, the aim was to identify the downstream signalling pathways of PGRMC1 by analysing the changes in the phosphoproteome following AG-205 treatment and PGRMC1 silencing. GO analysis revealed alterations to several key signalling pathways, including PI3K/AKT signalling, intracellular signalling by second messengers, signal transduction and diseases of signal transduction pathways. Moreover, it has been mechanistically confirmed that PGRMC1 directly interacts and regulates EGFR levels in lung and breast cancer cells.^18^ Similar studies have demonstrated that this interaction regulates tumour proliferation mainly through the phosphorylation of EGFR, AKT and ERK in colon cancer cells.^10^ Interestingly, silencing PGRMC1 in lung and breast cancer cells was found to decrease the expression of total EGFR.^18^ In the present study, GO analysis results suggest that PGRMC1 exerts its effects mainly through PI3K/AKT signalling. Consequently, the analysis focus shifted to identifying downstream signalling associated with these pathways following AG-205 treatment and PGRMC1 silencing. The obtained results indicated that, in ZR-75-1 cells, there was decreased phosphorylation of AKT at Thr246, Thr308, Tyr326, Tyr474 and mTOR Thr2446 and downstream targets P70S6K, Ser371 and Thr421. In MDA-MB-468 cells, we observed a decrease in AKT Ser124, Thr450, Tyr326 and mTOR Ser2448, and downstream P70S6K, Ser371 and Ser424. In addition, we also observed a decrease in EGFR Thr678, Thr693, Tyr1092, Tyr1110 and Tyr1197 reinforcing the GO analysis results. Western blot analysis demonstrated increased expression of phosphorylation of EGFR Ser1070 in MDA-MB-468 cells, following both treatments. Phosphorylation of EGFR Ser1070 has been demonstrated to cause EGFR to be internalised and degraded.^21,32–34^ Because PGRMC1 is known to interact with EGFR in cellular membranes, we hypothesised that AG-205 destabilises the PGRMC1/EGFR axis, leading to the downregulation of EGFR without disrupting the expression of PGRMC1.

Interestingly, IF performed following both treatments demonstrated a translocation of EGFR from the cell membrane to the cytoplasm in MDA-MB-468 cells, while EGFR was undetectable in ZR-75-1 cells. We speculate that in MDA-MB-468 cells, phosphorylation of EGFR Ser1070 causes EGFR to translocate from the cell membrane to the cytoplasm to be degraded, and relies heavily on the presence of EGFR for cell survival, as it lacks both ER and PR.^35–37^ On the other hand, ZR-75-1 can rely on both ER and PR, and can, therefore, internalise and degrade EGFR faster and more readily.^38,39^ Moreover, phosphoproteome analysis of MCF10A breast cells, in which PGRMC1 overexpression was induced, showed changes to PI3K/AKT signalling in cancer and signalling by receptor tyrosine kinases. Alterations in expression of cell survival genes (*EGFR, AKT1, PI3KCD, PTEN* and *mTOR*) were also observed. Specifically, a decrease in PTEN expression was observed, while phosphorylation of AKT Ser473, mTOR Ser2448 and EGFR Tyr1068 increased. PTEN is a potent tumour suppressor upstream of the PI3K pathway, and its activation is probably one of the reasons for the decrease in the PI3K/AKT pathway.^40–42^

In summary, we demonstrated that PGRMC1 activates the PI3K/AKT/mTOR and EGFR signalling pathways associated with cell survival in ER-positive and TNBC cells. We thus posit that PGRMC1 overexpression in breast cancer cells could promote cell survival by interacting with EGFR in the cell membrane. Its oncogenic role relies on the potential activation of downstream targets PI3K/AKT/mTOR, and deactivation of the upstream tumour suppressor PTEN. Our work thus provides evidence indicating that PGRMC1 could be a potential oncogene, as it has the capacity for regulating cell survival pathways in breast cancers.

## Supplementary information

Supplementary File

Data Set 1

Data Set 2

Data Set 3

## Data Availability

All data generated or analysed during this study are included in this published article [and its Supplementary information files].
