# Supplementary material for: Progesterone receptor membrane component 1 promotes the growth of breast cancers by altering the phosphoproteome and augmenting EGFR/PI3K/AKT signalling
Source: Br J Cancer. 2020 Jul 24;123(8):1326–35. doi: 10.1038/s41416-020-0992-6 (PMC7553958; doi:10.1038/s41416-020-0992-6)
Supplement: Supplementary file 1 — Supplementary File [file 41416_2020_992_MOESM1_ESM.pdf]

Supplementary material.

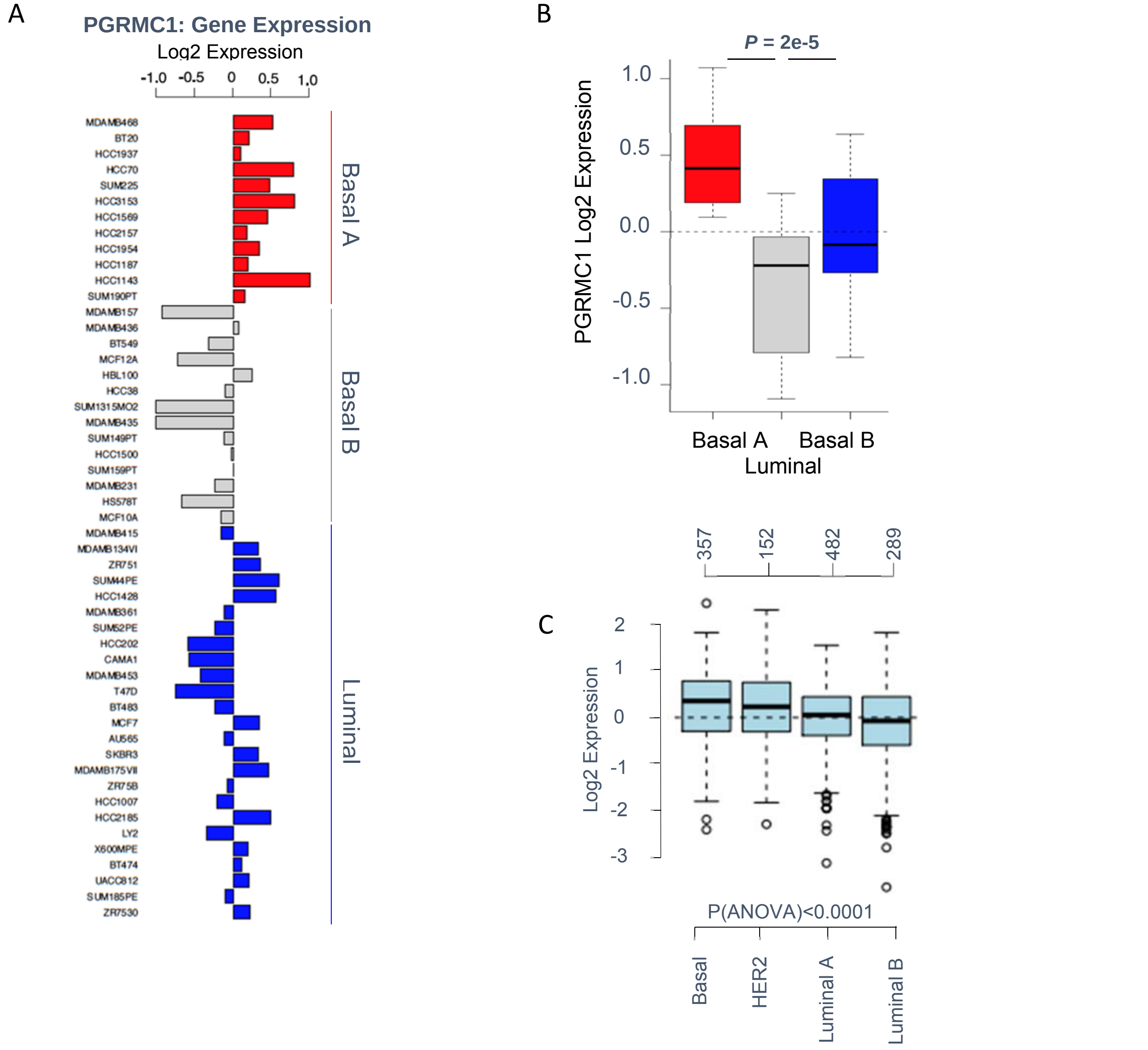

Fig. S1. **PGRMC1 is highly expressed in ER-positive and TNBCs.**

A. available gene expression repository, Gene expression-based Outcome for Breast Cancer (GOBO) was analyzed to observe PGRMC1 relative mRNA expression from a panel of 51 malignant and non-malignant breast cells, Basal A, B and Luminal epithelial cells. B. Box-plots displaying distribution of data the 51 breast cells. C. Box-plots displaying PGRMC1 expression in Basal (n=357), HER2 (n=152), Luminal A (n=482) and B (n=289) malignant breast tissue (n=number of tissue samples).

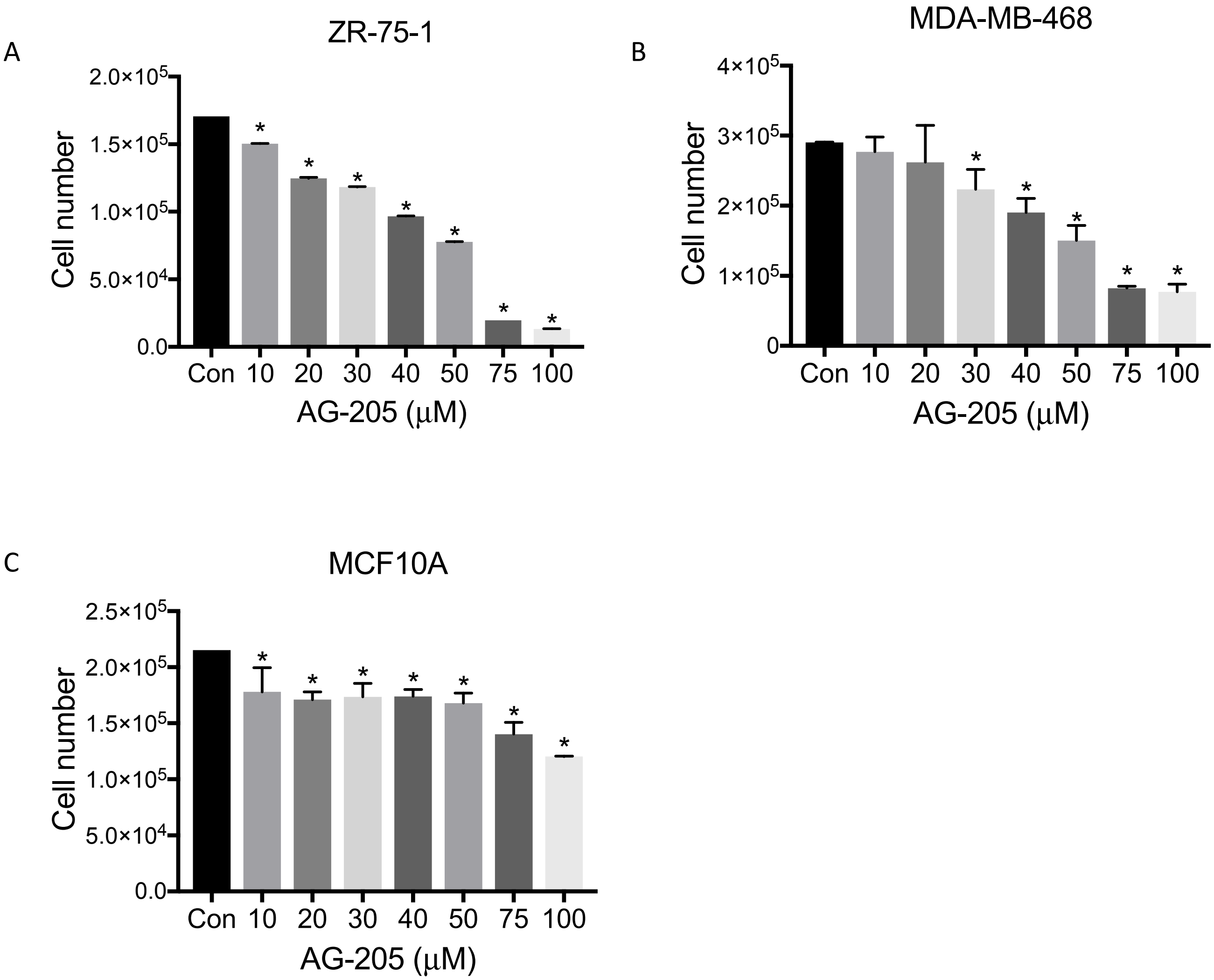

Fig. S2. **AG-205 selectively inhibits ER-positive and TNBC cell growth and survival.**

A. Dose dependent cell proliferation by hemocytometer following trypan blue staining of ZR-75-1, MDA-MB-468 breast cancer cells and normal breast MCF10A cells following 10, 20, 30, 40, 50, 75 and 100μM AG-205 treatment for 24 hours.

A

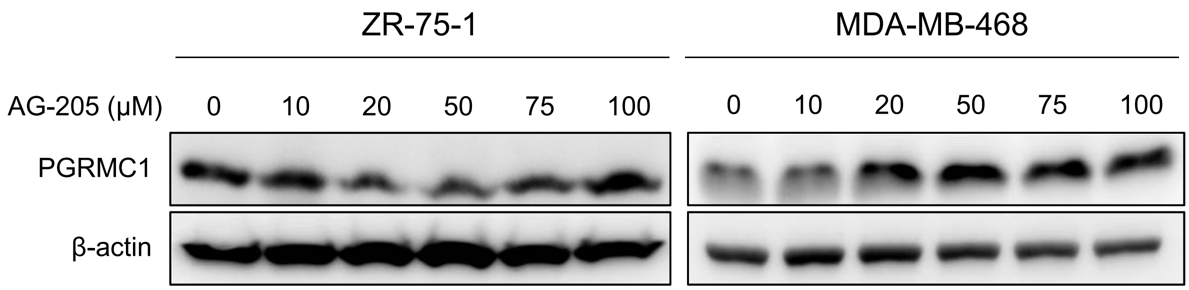

B

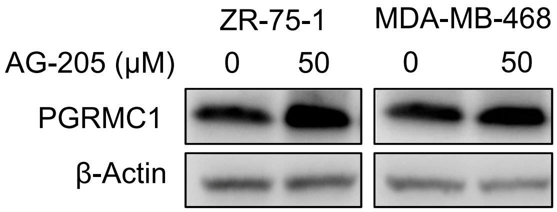

Fig. S3. **AG-205 has minimal effects on PGRMC1 expression.**

A. PGRMC1 expression by western blot following 10, 20, 50, 75 and 100 µM AG-205 treatment for 24 hours in ZR-75-1 and MDA-MB-468 breast cancer cells. B. Expression of PGRMC1 following 50 µM AG-205 treatment in ZR-75-1 and MDA-MB-468 breast cancer cells.

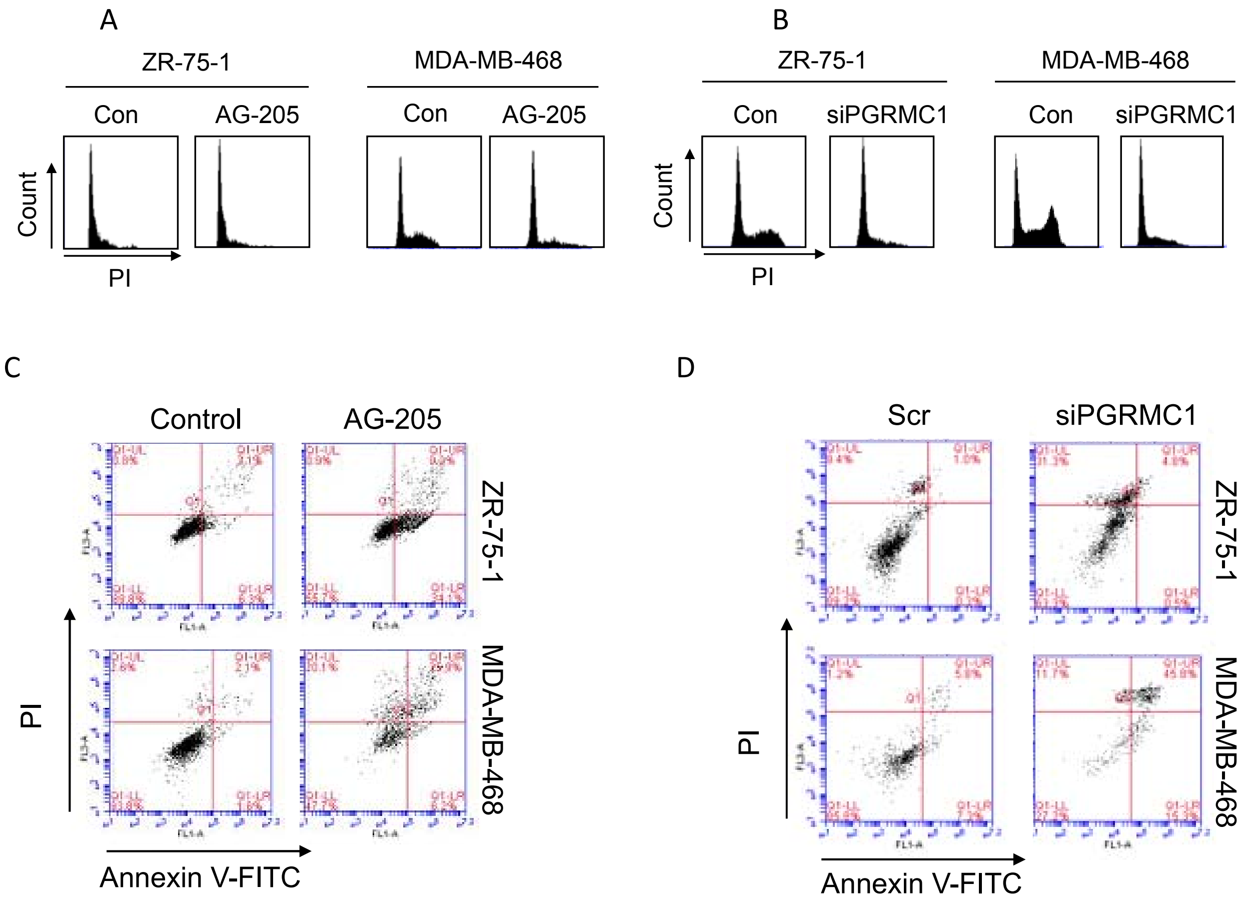

Fig. S4. **PGRMC1 signal inhibition and silencing promote cell cycle arrest and apoptosis.** A. Cell cycle analysis by flow cytometry following 50µM AG-205 treatment or B. silencing PGRMC1 in ZR-75-1 and MDA-MB-468 cells. C. Apoptosis analysis by flow cytometry following 50µM AG-205 treatment or C. silencing PGRMC1 in ZR-75-1 and MDA-MB-468 cells.

ZR-75-1

A

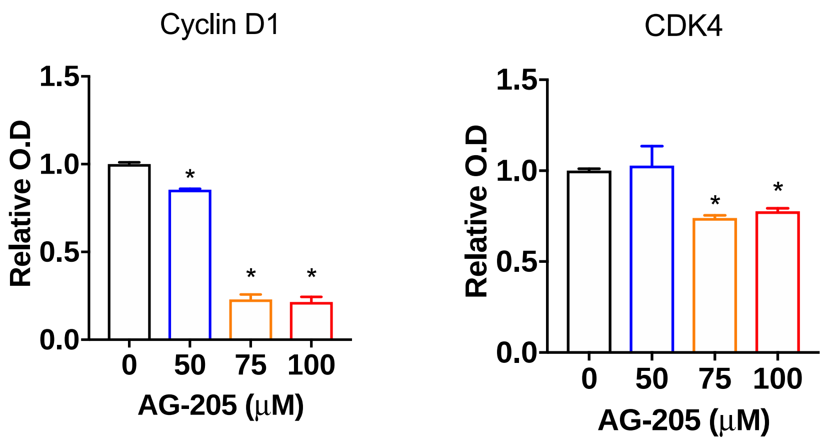

MDA-MB-468

B

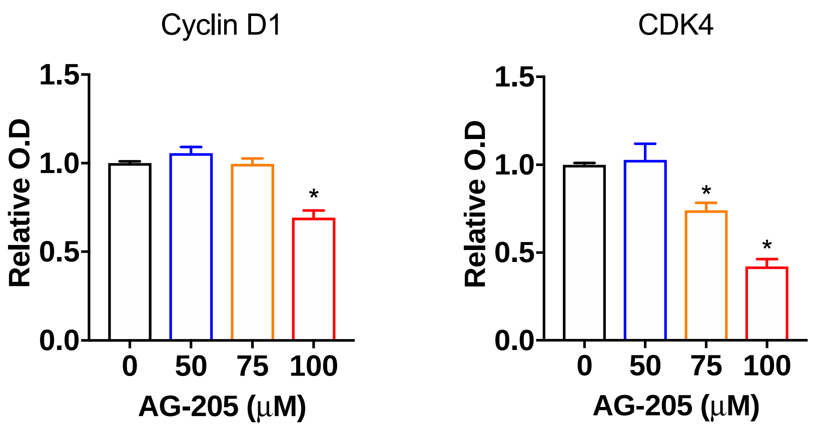

Fig. S5. **Densitometric analysis of cell cycle proteins following AG-205 treatment.**

A-B. Densitometric analysis of cell cycle proteins, Cyclin D1 and CDK4 following 50, 75 and 100μM of AG-205 treatment in ZR-75-1 and MDA-MB-468 cells.

ZR-75-1

A

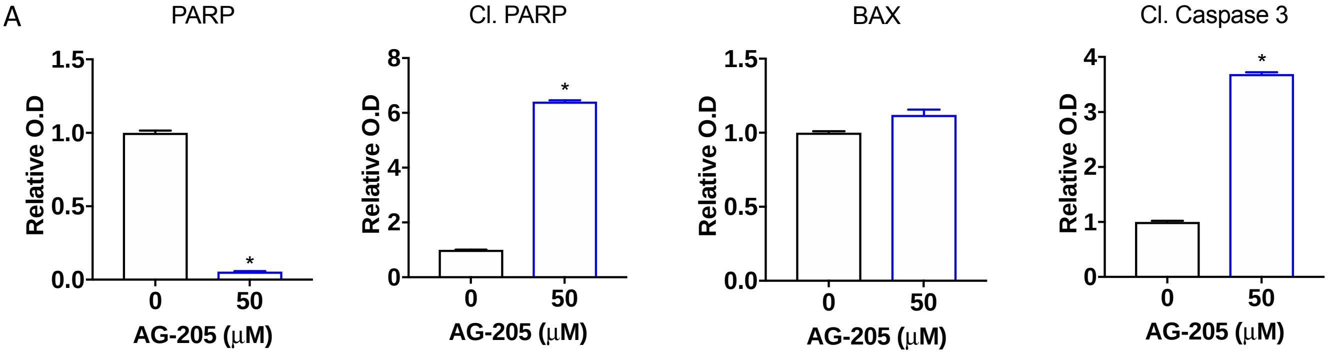

MDA-MB-468

B

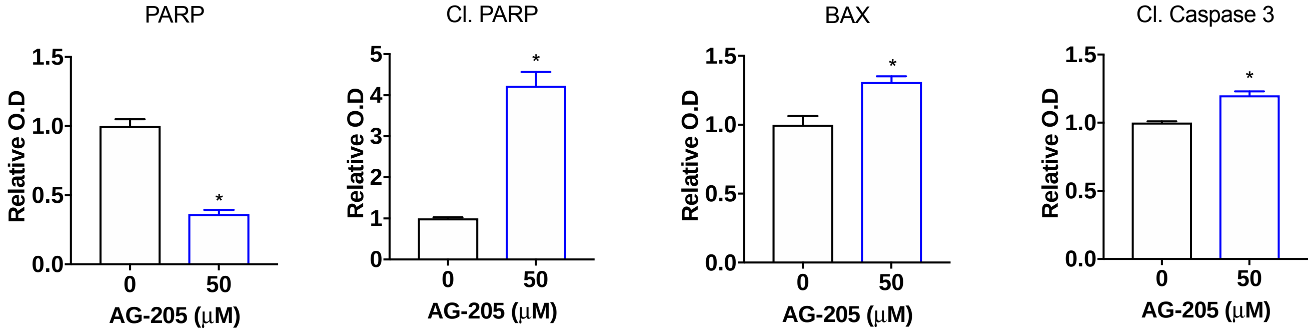

Fig. S6. **Densitometric analysis of apoptotic proteins following AG-205 treatment.**

A-B. Densitometric analysis of apoptotic proteins, PARP, Cl. PARP, BAX and Cl. Caspase 3 following 50μM treatment of AG-205 in ZR-75-1 and MDA-MB-468 cells.

A

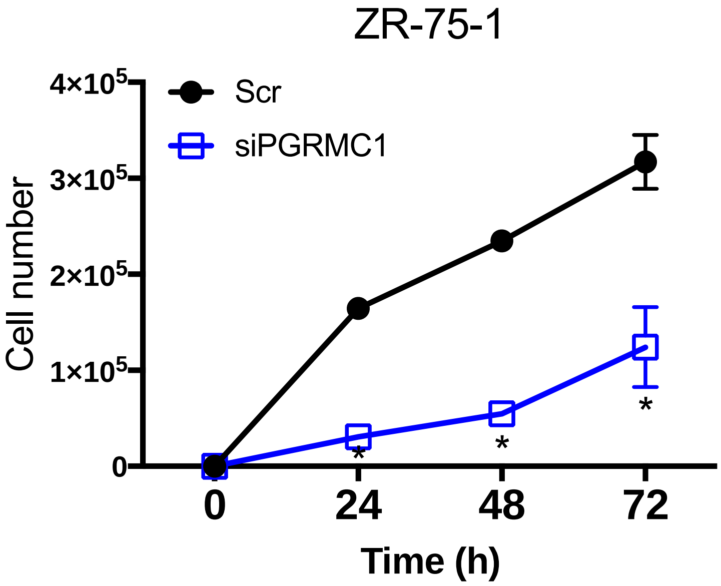

B

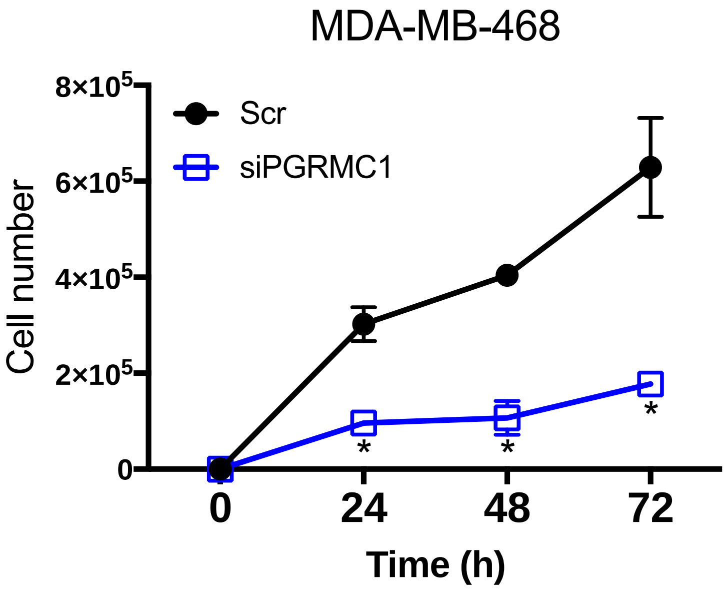

Fig S7**. Silencing PGRMC1 inhibits growth and survival of breast cancer cells**

A – B. Time dependent cell proliferation by hemocytometer following trypan blue staining following PGRMC1 silencing by siRNA in ZR-75-1 and MDA-MB-468 cells.

ZR-75-1

A

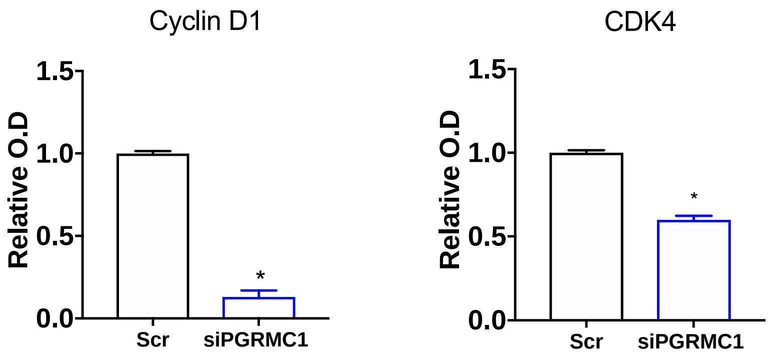

MDA-MB-468

B

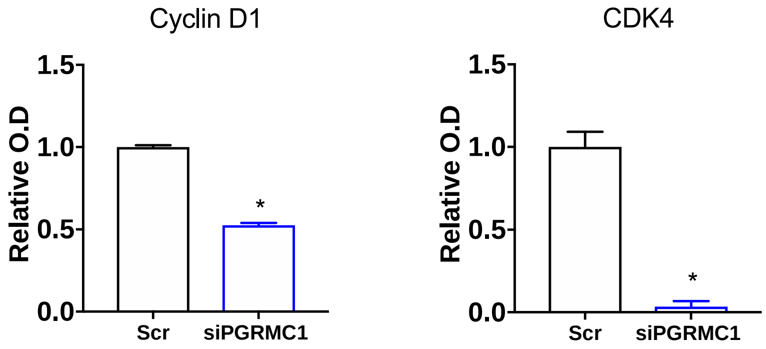

Fig. S8. **Densitometric analysis of cell cycle proteins following PGRMC1 silencing.**

A-B. Densitometric analysis of cell cycle proteins, Cyclin D1 and CDK4 following PGRMC1 silencing in ZR-75-1 and MDA-MB-468 cells.

ZR-75-1

A

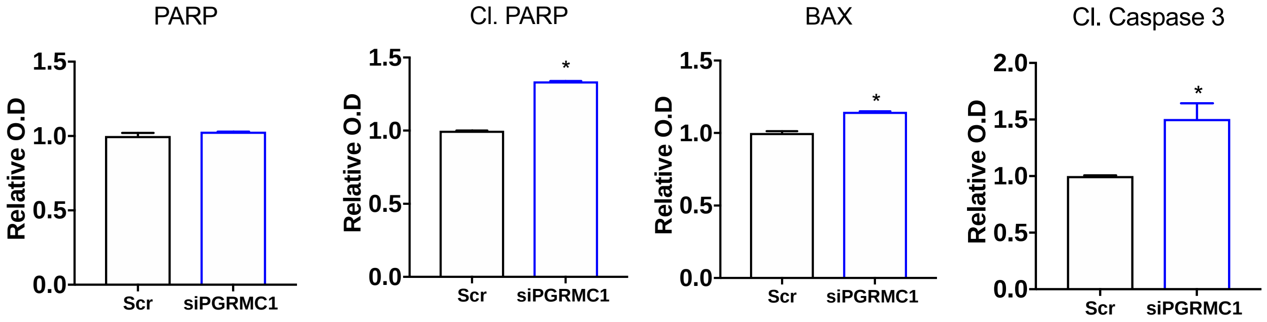

MDA-MB-468

B

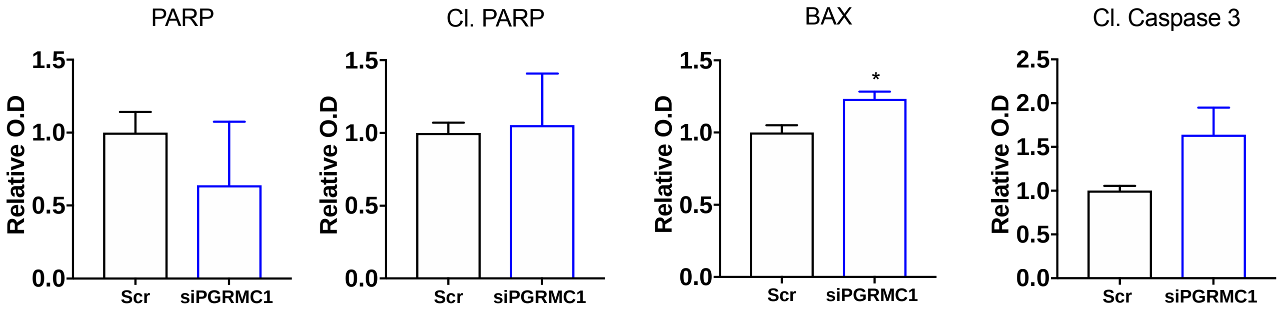

Fig. S9. **Densitometric analysis of apoptotic proteins following PGRMC1 silencing.**

A-B. Densitometric analysis of apoptotic proteins, PARP, Cl. PARP, BAX and Cl. Caspase 3 following PGRMC1 silencing in ZR-75-1 and MDA-MB-468 cells.

A **ZR-75-1**

AG-205/siPGRMC1

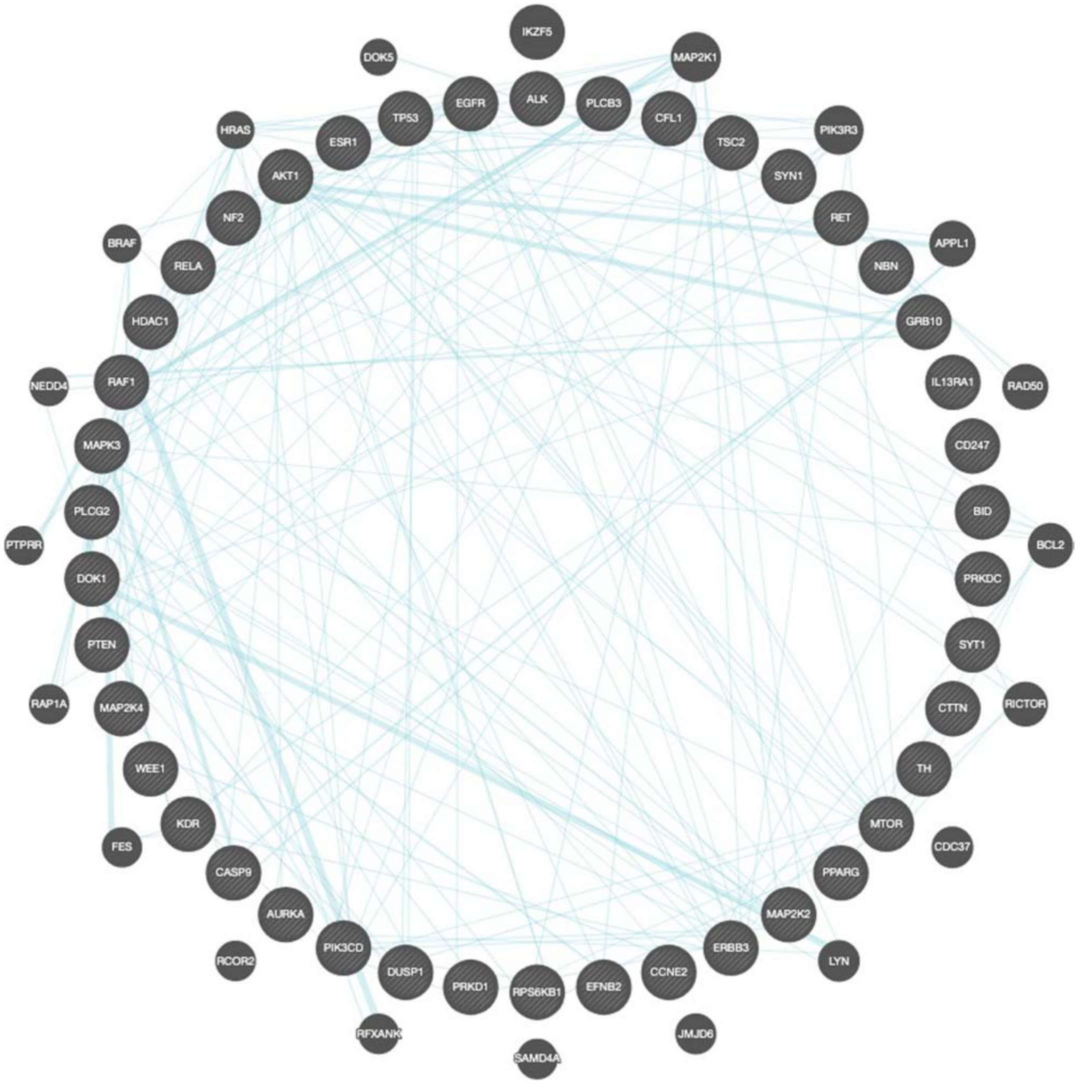

Fig. S10. **Phospho-proteome analysis connects PGRMC1 signaling to breast cancer proliferation.**

A. Network analysis of commonly enriched genes following both 50μM AG-205 and PGRMC1 silencing in ZR-75-1 cells. The genes observed exhibited interactions with cell proliferative and cell survival genes.

A **MDA-MB-468**

AG-205/siPGRMC1

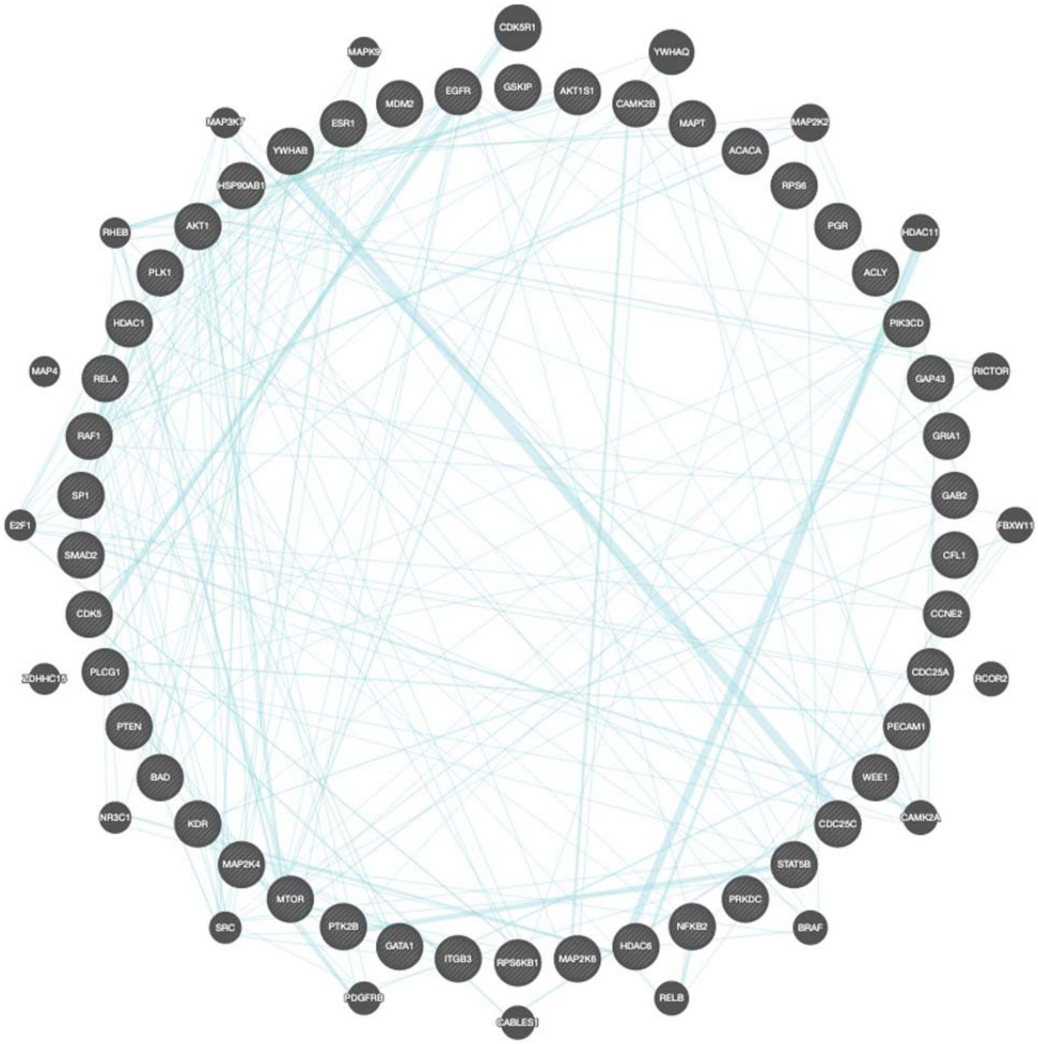

Fig. S11. **Phospho-proteome analysis connects PGRMC1 signaling to breast cancer proliferation.**

B. Network analysis of commonly enriched genes following both 50µM AG-205 and PGRMC1 silencing in MDA-MB-468 cells. The genes observed exhibited interactions with cell proliferative and cell survival genes.
